# Effectiveness and adverse events of plasmapheresis and immunoglobulin G infusion in patient with resistant chronic urticaria and angioedema - a case report

**DOI:** 10.1186/2045-7022-5-S1-P8

**Published:** 2015-03-11

**Authors:** Izabela Kuprys-Lipinska, Paulina Korczynska, Piotr Kuna

**Affiliations:** 1 Medical University of Lodz, Internal Medicine, Asthma and Allergy, Lodz, Poland

## Background

In most cases of chronic urticaria, non--sedating anti-histamine drugs (nsAnti-H1) control the symptoms, and the remission is obtained after 1--5 years.

## Aim

Here is presented a case of 54 years old woman with the history of severe chronic urticaria from over 30 yrs resistant to nsAnti-H1 and treated with plasmapheresis and intravenous immunoglobulin G.

## Results

In this patient urticaria with angioedema eruptions were induced by cold, pressure, NSAID, antibiotics, but mainly appeared spontaneous, and were aggravated before menstruation. She was twice hospitalized due to the severe angioedema. nsAnti-H1 in standard and high doses were ineffective, and caused the drowsiness. Concomitant treatment with LTRA, anti-H2 and sAnti-H1 as well as diet were also ineffective. Only high doses of oral corticosteroids (oCS) partly controlled the clinical symptoms. Autologous serum skin test was positive, skin biopsy revealed oedema of derma and infiltration by eosinophils and lymphocytes. She had also the history of thyroid gland disorder. Due to autoimmunologic background plasmapheresis was applied. During first cycle adverse event (AE) -- eruption of urticaria with angioedema developed. AE was treated with intravenous CS (ivCS) and Anti-H1. Next cycles with CS premedication proceeded without AE. Remission of urticaria lasted 6 month, and then symptoms relapsed. Second series of plasmapheresis and plasmapheresis booster preceeded CS premedication passed without AE, but during intravenous immunoglobulin G infusion, applied for continuation, urtical reaction with angioedema again appeared and disappeared after treatment with ivCS. Patient was again free from symptoms through 6 months. Then symptoms turn back but they could be control by low doses of oCS with nsAnti-H1. In 2008 she had hysterectomy due to cervical cancer (in situ). Nowadays she is still symptomatic despite treatment but symptoms are mild and tolerated. She refused anti-IgE therapy due to cancerophobia.

## Conclusion

In some patients suffering from severe chronic urticaria a compromise in symptoms control and therapy intensity need to be obtain and accepted by a patient and a physician.

## Consent

Written informed consent was obtained from the patient for publication of this abstract and any accompanying images. A copy of the written consent is available for review by the Editor of this journal.

